# Comparison of Daily Routines Between Middle-aged and Older Participants With and Those Without Diabetes in the Electronic Framingham Heart Study: Cohort Study

**DOI:** 10.2196/29107

**Published:** 2022-01-07

**Authors:** Yuankai Zhang, Chathurangi H Pathiravasan, Michael M Hammond, Hongshan Liu, Honghuang Lin, Mayank Sardana, Ludovic Trinquart, Belinda Borrelli, Emily S Manders, Jelena Kornej, Nicole L Spartano, Christopher Nowak, Vik Kheterpal, Emelia J Benjamin, David D McManus, Joanne M Murabito, Chunyu Liu

**Affiliations:** 1 Department of Biostatistics Boston University School of Public Health Boston, MA United States; 2 Boston University's and National Heart, Lung, and Blood Institute's Framingham Heart Study Framingham, MA United States; 3 Section of Computational Biomedicine, Department of Medicine Boston University School of Medicine Boston, MA United States; 4 Cardiology Division, Department of Medicine University of California San Francisco San Francisco, CA United States; 5 Center for Behavioral Science Research, Henry M. Goldman School of Dental Medicine Boston University Boston, MA United States; 6 Section of Endocrinology, Diabetes, Nutrition, and Weight Management Boston University School of Medicine Boston, MA United States; 7 Care Evolution Ann Arbor, MI United States; 8 Section of Preventive Medicine and Epidemiology and Cardiovascular Medicine, Department of Medicine Boston University School of Medicine Boston, MA United States; 9 Department of Epidemiology Boston University School of Public Health Boston, MA United States; 10 Cardiology Division, Department of Medicine University of Massachusetts Medical School Worcester, MA United States; 11 Department of Quantitative Health Sciences University of Massachusetts Medical School Worcester, MA United States; 12 Section of General Internal Medicine, Department of Medicine Boston University School of Medicine Boston, MA United States

**Keywords:** diabetes, mobile health, smartwatch, daily physical activities, daily routine pattern, sleep, step counts, diabetes self-management, mobile phone

## Abstract

**Background:**

Daily routines (eg, physical activity and sleep patterns) are important for diabetes self-management. Traditional research methods are not optimal for documenting long-term daily routine patterns in participants with glycemic conditions. Mobile health offers an effective approach for collecting users’ long-term daily activities and analyzing their daily routine patterns in relation to diabetes status.

**Objective:**

This study aims to understand how routines function in diabetes self-management. We evaluate the associations of daily routine variables derived from a smartwatch with diabetes status in the electronic Framingham Heart Study (eFHS).

**Methods:**

The eFHS enrolled the Framingham Heart Study participants at health examination 3 between 2016 and 2019. At baseline, diabetes was defined as fasting blood glucose level ≥126 mg/dL or as a self-report of taking a glucose-lowering medication; prediabetes was defined as fasting blood glucose level of 100-125 mg/dL. Using smartwatch data, we calculated the average daily step counts and estimated the wake-up times and bedtimes for the eFHS participants on a given day. We compared the average daily step counts and the intraindividual variability of the wake-up times and bedtimes of the participants with diabetes and prediabetes with those of the referents who were neither diabetic nor prediabetic, adjusting for age, sex, and race or ethnicity.

**Results:**

We included 796 participants (494/796, 62.1% women; mean age 52.8, SD 8.7 years) who wore a smartwatch for at least 10 hours/day and remained in the study for at least 30 days after enrollment. On average, participants with diabetes (41/796, 5.2%) took 1611 fewer daily steps (95% CI 863-2360; *P*<.001) and had 12 more minutes (95% CI 6-18; *P*<.001) in the variation of their estimated wake-up times, 6 more minutes (95% CI 2-9; *P*=.005) in the variation of their estimated bedtimes compared with the referents (546/796, 68.6%) without diabetes or prediabetes. Participants with prediabetes (209/796, 26.2%) also walked fewer daily steps (*P*=.04) and had a larger variation in their estimated wake-up times (*P*=.04) compared with the referents.

**Conclusions:**

On average, participants with diabetes at baseline walked significantly fewer daily steps and had larger variations in their wake-up times and bedtimes than the referent group. These findings suggest that modifying the routines of participants with poor glycemic health may be an important approach to the self-management of diabetes. Future studies should be designed to improve the remote monitoring and self-management of diabetes.

## Introduction

### Background

Diabetes affects millions of people worldwide. It is estimated that >30 million people currently have diabetes in the United States, with that number expected to rise to 44.1 million by 2034 [1]. From 2015 to 2016, the annual diabetes-related health care costs increased from US $43.9 billion to US $51.5 billion in the United States [1]. As diabetes is associated with increased morbidity and mortality, and it ultimately predisposes the patient to heart disease, stroke, and kidney disease [2], lifestyle management should be a fundamental aspect of diabetes care in addition to medication treatment [3]. Healthy eating, more exercise, a regular sleep habit, smoking cessation, and stress management are 5 essential factors in diabetes lifestyle management [4]. Among these 5 factors, lack of exercise is a significant predictor of incident diabetes, which is independent of obesity [5,6]. In contrast, lack of exercise leads to obesity and being overweight, and excess body fat results in insulin resistance [7,8]. Therefore, the adoption and maintenance of physical activity are critical for the management of healthy weight and blood glucose levels in individuals with poor glycemic health [4,9]. In addition, previous studies have shown that sleep disturbance, which is similar to several traditional risk factors, is also a significant risk factor for diabetes [10], and chronic circadian disruption caused by sleep–wake cycle irregularities increases the risk of metabolic syndrome and diabetes [11,12]. Given the important roles of exercise and sleep in diabetes risk, self-monitoring of daily routine patterns may motivate people to adopt and maintain healthy lifestyles and, therefore, improve glycemic health in participants with diabetes.

Most previous studies that investigated daily routine patterns, for example, sleep patterns and physical activities, collected data using traditional epidemiological research methods such as self-reported questionnaires or surveys [13-15]. These traditional methods often collect data at a single time point or a few time points. In addition, these traditional methods are costly (eg, in-person interviews with a large number of participants) and are more likely to be subject to recall bias [16,17]. In the past few years, an accelerometer or pedometer has been used to access daily step count, although most accelerometer or pedometer studies only collect step counts within a few days or a couple of weeks [18]. Mobile health (mHealth) is an emerging technology that is increasingly being used worldwide [19]. mHealth enables continuous ambulatory monitoring of the health status and daily activities of users [20], making it possible to collect reliable daily routine patterns in large cohort studies with long-term follow-up. One of the early application areas of mHealth is diabetes remote monitoring and self-management [21]. mHealth provides a convenient and effective way of engaging people in digital diabetes care and self-management, including physical exercise management, insulin dosage calculation, and so forth [22-24]. However, the application of mHealth in diabetes self-management in community-based cohorts remains to be studied.

### Objective

The electronic Framingham Heart Study (eFHS) is a cohort study in which participants were provided smartwatches and instructed to wear them each day. mHealth technology has allowed the participants in the eFHS to document their daily routine patterns in a relatively inexpensive and convenient way. On the basis of previous findings, this study aims to investigate the associations of daily routine patterns with diabetes status in the eFHS. We include 796 participants returning daily steps and heart rates for at least 1 to 36 months via smartwatches (average return 9.6 months). The sleep routine patterns included several smartwatch-derived proxy measures for wake-up times, bedtimes, and sleep durations. We perform association analyses of daily routine patterns (step counts and sleep pattern variables) with diabetes status. We hypothesize that, compared with the referents, participants with diabetes and prediabetes walk fewer steps per day and have higher variability in their daily sleep patterns, which were measured by the smartwatch.

## Methods

### Study Sample

The eFHS is nested in the Framingham Heart Study (FHS), a community-based, prospective study that was initiated in 1948 in the town of Framingham, Massachusetts [25-27]. The study sample included participants in three cohorts—the third-generation cohort (generation 3), a cohort of multiple ancestries (omni 2), and a cohort of new offspring spouses (NOS)—who attended their third research center examination in person [25]. In the eFHS, we developed a smartphone app that included electronic consent and health questionnaires and integrated both a wireless blood pressure cuff and a smartwatch. The eFHS recruited approximately 2100 FHS participants who owned a smartphone (iPhone 4S or newer iPhone with at least an iOS 8.2 or Android phone), attended an FHS health examination in person between 2016 and 2019, and consented to participate in eFHS. For this study, we included participants with iPhones, who were offered a study smartwatch (Apple Watch v.0 [Apple Inc]) to record vital data (step and heart rate data). The participants who owned an Apple Watch were allowed to use their own smartwatches. The participants in the eFHS were invited to download the eFHS smartphone app and were provided a written protocol that included information on how to download the app, enter the registration information, sign the consent forms, enable notifications on their phones, and set up the smartwatches. Daily battery charging was needed for this version of the Apple Watch. To maximize the data collection during the daytime, the participants were instructed to wear the smartwatch after waking up in the morning and take off the smartwatch at bedtime to charge the smartwatch battery.

A total of 1127 participants who enrolled in the eFHS chose to use a smartwatch (1010/1127, 89.62% from generation 3, 17/1127, 1.51% from NOS, and 100/1127, 8.87% from omni 2). These participants returned heart rate and step data from the smartwatch for up to 3 years. Participants who developed cardiovascular disease may have had severe health issues and may have confounded the association analyses in this study. The main aim of this study was to access long-term daily routine patterns in relation to diabetes status. Therefore, of the 1127 participants, we excluded a total of 331 (29.37%) participants (Multimedia Appendix 1). The excluded participants had cardiovascular conditions at the third FHS health examination (39/331, 11.8%), wore the smartwatch for <10 hours per day (43/331, 12.9%; see the *Outcome Variables* section), or returned smartwatch data for <30 days (249/331, 75.2%).

The Boston University Medical Campus institutional review board reviewed and approved the study, and all participants provided informed consent via the eFHS.

### Outcome Variables

**Figure 1:**
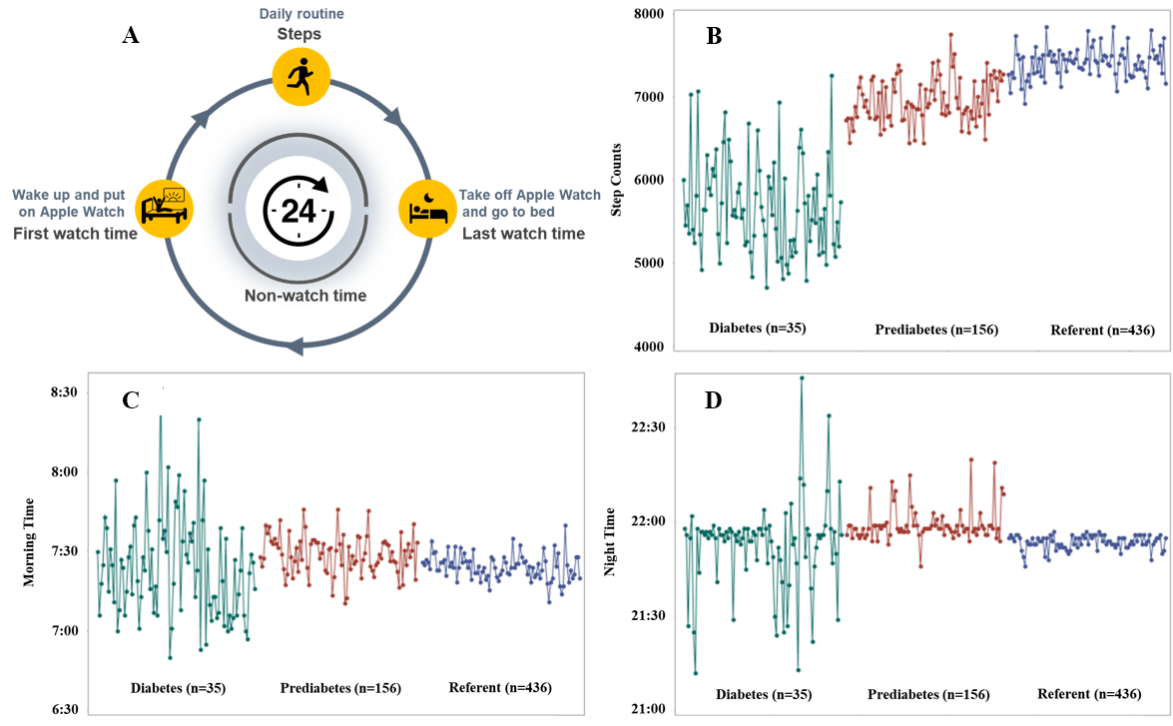
Daily routine pattern and observational measures of smart watch variables within 90 days of follow-up. (A) Variables of daily routine pattern of a participant. (B) The median values of daily step counts from participants in 3 diabetes categories within 90 days. (C) The median values of first watch time from participants in 3 diabetes categories within 90 days. (D) The median values of last watch time from participants in 3 diabetes categories within 90 days. The y-axis is the median value of daily steps (B), first watch time (C), and last watch time (D) using a 24-hour format.

The participants in the eFHS were instructed to wear the smartwatch after waking up in the morning and remove the watch before bedtime. *Watch time* was the time during which any heart rate or step data were detected by the smartwatch. Therefore, on a calendar day, it was reasonable to assume that the first watch time (referred to as first watch time) reflected a participant’s wake-up time. Similarly, the last watch time (referred to as last watch time) reflected a participant’s bedtime (Figure 1**)**. However, it was difficult to determine whether a time detected by the watch was from the previous day or the following day if it occurred very late in the night or early in the morning (eg, after 12 AM and before 4 AM). We examined the distribution of the first watch time and last watch time on all calendar days. We found that 86% of the first watch time occurred after 4 AM on any calendar day and that 90% of the last watch time occurred after 7 PM on any calendar day (Multimedia Appendices 2 and 3). Therefore, we identified the first watch time if it occurred between 4 AM and noon (12 PM) on a given day. We excluded any person’s day if the participant’s first watch time was beyond this time interval. Similarly, we identified the last watch time if it occurred between 7 PM and midnight (ie, 12 AM), provided that a participant’s first watch time occurred after 4 AM the following day. We excluded any person’s day if the last watch time was beyond this time interval. The identified first watch time and last watch time were used as proxy measures to estimate wake-up times and bedtimes, respectively, on each day. For any 2 consecutive watch days, we calculated the non–watch time as the total time between the last watch time on a given watch day and the first watch time on the next watch day. Non–watch time was used as a proxy measure to estimate a participant’s time spent asleep. To study the irregularity of daily routines for every participant, we calculated the mean value of the first watch time using the data collected during the entire eFHS study.

Next, we calculated the absolute deviations between the observed first watch times and the mean value on all follow-up days for each participant. Similarly, we calculated the absolute deviations for the last watch times and non–watch times for each participant on all follow-up days. The absolute deviations of the times for the 3 watch variables reflected the intraindividual variation of each watch time variable during the entire follow-up period. These repeated absolute deviations of the 3 watch time variables were used as outcome variables in association analyses with diabetes status.

In addition, we used repeated daily step counts collected from the smartwatch as an outcome variable for physical activity in association analyses with diabetes. Daily steps largely reflect people’s routine daily physical activities, and previous studies support the use of daily step count as a measurement for assessing the association of physical activity with diabetes [13,28]. The Apple Watch used a built-in accelerometer to track users’ wrist motion and then estimated the step counts [29]. We used repeated daily step counts collected from the smartwatch as an outcome variable for physical activity in association analyses with diabetes.

### Diabetes, Prediabetes, and Covariates at FHS Health Examination

At each health examination, blood samples were obtained after an overnight fast (approximately 10-12 hours), and plasma samples were immediately processed and kept at –80°C until assayed [25,30]. Glucose levels were measured in blood plasma [25,30]. We defined a 3-level categorical diabetes variable to classify participants with diabetes, prediabetes, or normal fasting blood glucose levels (ie, the referents). Diabetes was defined as a fasting blood glucose level ≥126 mg/dL or whether the participant was taking any blood glucose-lowering medications [31]. Prediabetes status was defined as a fasting blood glucose level of 100-125 mg/dL [31]. The referents were participants without diabetes or prediabetes. This 3-level diabetes category variable was used as the independent variable in all the statistical analyses.

### Statistical Analyses

The baseline characteristics of the participants were described as means and SDs for continuous variables and frequencies (percentages) for categorical variables. We further compared the proportions of the remaining eFHS participants with diabetes and prediabetes with the referents at 30-, 60-, 90-, and 180-day windows.

After exclusion, we included the rest of the observations from the remaining participants in the association analysis. We applied a linear mixed regression model to investigate the associations between the outcome variables and diabetes category variables. The outcome variables were the repeated daily absolute deviations of the 3 watch time variables and repeated daily step counts.

We conducted 3 models. In the primary model, model 1, the covariates included sex, age, and self-reported race or ethnicity. Model 2 included BMI (kg/m^2^) in addition to the covariates in model 1. Model 3 was further adjusted for current smoking and current alcohol consumption. Age, current smoking, and current alcohol consumption were collected in person during the third health examination.

In the analysis of steps as the outcome variable, we added the daily smartwatch wearing time as an additional covariate in the 3 models, as daily smartwatch wearing time was expected to be strongly associated with the number of daily steps.

All statistical analyses were performed using the SAS software (version 9.4; SAS Institute Inc). We used a 2-tailed *P*<.05 for significance.

To further investigate the daily step counts and the variation of the first watch time and last watch time from participants with diabetes and prediabetes versus referents during the study, we calculated and plotted the median value of each outcome variable per day from participants in each of the 3 diabetes categories. The number of participants (41/796, 5.2%) with diabetes was much smaller than the number of participants with prediabetes and the number of referents. To make a fair comparison, we performed a sampling procedure to randomly select 41 participants from the prediabetes and referent groups and plotted the median value of each outcome in the 3 diabetes categories.

## Results

### Characteristics of Study Participants

We excluded 331 participants (mean age 52.7, SD 8.5 years; 214/331, 64.7% women) who wore a smartwatch for <5 hours/day or remained in the study for <30 days. Of the 331 excluded participants, 19 (5.9%) had diabetes, 80 (24.6%) had prediabetes, and 226 (69.5%) had neither diabetes nor prediabetes (Multimedia Appendix 4). A total of 796 participants (710/796, 89.2% in generation 3, 12/796, 1.5% in NOS, and 74/796, 9.3% in omni 2) were included in the study. The median follow-up period of the participants in this study was 219 days (first quartile to third quartile: 109-377 days). Of the 796 participants, the study sample included 41 (5.2%) participants with diabetes, 209 (26.2%) with prediabetes, and 546 (68.6%) referents. The median follow-up duration for the participants in the 3 diabetes categories was not significantly different (Kruskal–Wallis test, *P*=.28). The participants with diabetes and prediabetes remained in the study for similar durations when we evaluated the 30-, 60-, 90-, and 180-day windows compared with the referents (Multimedia Appendix 5). For example, in the 90-day window, 85% (35/41) of participants with diabetes, 74.6% (156/209) of participants with prediabetes, and 79.9% (436/546) of referents remained in the study.

Compared with the referents (mean age 51.5, SD 8.7 years; 153/546, 28% men), participants with diabetes (mean age 57.4, SD 7.8 years; 24/41, 59% men) or prediabetes (mean age 55.3, SD 8.2 years; 125/209, 59.8% men) were older and tended to be men. In addition, as compared with referents, participants with diabetes or prediabetes had a higher BMI (33.4 kg/m^2^ and 30.2 kg/m^2^, respectively, vs 26.9 kg/m^2^), a lower proportion of graduate or professional degrees (10/41, 24% and 50/209, 23.9%, respectively, vs 182/546, 33.3%), and a higher proportion of current smoking (2/41, 5% and 14/209, 6.7%, respectively, vs 19/546, 3.5%; Table 1). Participants with diabetes were less likely to drink alcohol than the referents (27/41, 66% vs 453/546, 83%; Table 1).

**Table 1:** Characteristics of the electronic Framingham Heart Study participants in this study (N=796).

Characteristics		Diabetes^a^ (n=41)	Prediabetes^a^ (n=209)	Referents (n=546)
Age (years), mean (SD)		57.4 (7.8)	55.3 (8.2)	51.5 (8.7)
Women, n (%)		17 (41.5)	84 (40.2)	393 (72)
Alcohol drinking (yes), n (%)		27 (65.9)	175 (83.7)	453 (83)
Smoking (yes), n (%)		2 (4.9)	14 (6.7)	19 (3.5)
**Education, n (%)**				
	High school or less	7 (17.1)	21 (10)	33 (6.1)
	Completed some college	13 (31.7)	51 (24.4)	116 (21.2)
	Bachelor’s degree	11 (26.8)	86 (41.1)	214 (39.2)
	Graduate or professional degree	10 (24.4)	50 (23.9)	182 (33.3)
BMI (kg/m^2^), mean (SD)		33.4 (6.4)	30.2 (5.0)	26.9 (5.1)
Daily step (step counts), mean (SD)		6216 (3634)	7980 (3851)	8120 (3902)
Variation of first watch time^b^, mean (SD)		67 (57)	58 (53)	57 (51)
Variation of last watch time^b^, mean (SD)		52 (39)	46 (37)	46 (37)
Variation of non–watch time^b^, mean (SD)		77 (65)	66 (57)	66 (57)

^a^Diabetes was defined as fasting blood glucose ≥126 mg/dL or use of blood glucose-lowering medications. Prediabetes status was defined as a fasting blood glucose value between 100 and 126 mg/dL.

^b^Refer to Figure 1A and the *Methods* section for definitions. The unit for variation was minute.

We also compared the median daily smartwatch wearing time for participants in the 3 diabetes categories. The median daily watch-wearing time was the same: 14 hours (first quartile to third quartile: 13-15 hours; Kruskal–Wallis test for median daily smartwatch wearing times, *P*=.11) for the participants in all 3 groups.

We further compared the characteristics of participants in eFHS with the rest of the participants who were not enrolled in the eFHS but attended the third in-person FHS health examination. On average, the participants in this study were younger (mean age 52.8, SD 8.7 years) and had a better education (242/796, 30.4% had graduate or professional degrees) than those who were not in eFHS (mean age 56.8, SD 9.6 years; 252/1500, 16.8% had graduate or professional degrees). In addition, the eFHS participants appeared to be healthier. For example, this study included 5.2% (41/796) of participants with diabetes. In contrast, the participants who did not participate in the eFHS included 186 (186/1500, 12.4%) participants with diabetes (Multimedia Appendix 4).

### Association Analyses of Daily Steps and Diabetes Status

We first visualized the median daily step count between the participants with diabetes and the referents at the 90-day window (Figure 1B and Multimedia Appendix 6). The median daily step counts were between 4500 and 7200 for participants with diabetes. In contrast, the median daily step counts were between 6500 and 8000 for the referents (Figure 1B). We further performed association analyses to quantify the associations. On average, the participants with diabetes took 1611 fewer daily steps (95% CI 863-2360; *P*<.001) compared with referents, adjusting for age, sex, race, and daily watch-wearing time (Table 2). The participants with prediabetes took 392 fewer daily steps (95% CI 13-770; *P*=.04) compared with the referents. Adjusting for BMI in addition to age, sex, race, and daily watch-wearing time, the association between diabetes categories and average daily steps was greatly attenuated. In model 2, the participants with diabetes walked 773 fewer steps (95% CI 67-1479; *P*=.03) compared with referents (Table 2). The difference in the number of steps became nonsignificant between the participants with prediabetes and the referents after including BMI as an additional covariate (Table 2). Further adjustment for alcohol consumption and smoking as additional covariates slightly attenuated the associations between the diabetes categories and average steps (Table 2). In model 3, the participants with diabetes walked 799 fewer steps (95% CI 94-1503; *P*=.03) compared with referents (Table 2). To investigate whether the follow-up duration may confound the association between step counts and diabetes status, we included the number of follow-up days as an additional covariate in model 1 for a sensitivity analysis. We observed a minimum change in the regression estimate for daily step counts as the outcome variable (Multimedia Appendix 7).

**Table 2:** Association between diabetes categories and daily routine patterns measured by the smartwatch.

Outcome and diabetes categories		Model 1^a^		Model 2^b^		Model 3^c^	
		Mean differences (95% CI)	*P* value	Mean differences (95% CI)	*P* value	Mean differences (95% CI)	*P* value
**Daily steps^d^**							
	Referent	Reference	N/A^e^	Reference	N/A	Reference	N/A
	Prediabetes	−392 (−770 to −13)	.04	−11 (−380 to 359)	.96	−2 (−371 to 367)	.99
	Diabetes	−1611 (−2360 to −863)	<.001	−773 (−1479 to −67)	.03	−799 (−1503 to −94)	.03
**Variation of first watch time^f^**							
	Referent	Reference	N/A	Reference	N/A	Reference	N/A
	Prediabetes	3 (0 to 7)	.048	3 (−1 to 6)	.10	3 (−1 to 6)	.12
	Diabetes	12 (6 to 18)	<.001	11 (4 to 17)	.001	10 (4 to 17)	.002
**Variation of last watch time^f^**							
	Referent	Reference	N/A	Reference	N/A	Reference	N/A
	Prediabetes	1 (−1 to 3)	.16	1 (−1 to 3)	.29	1 (−1 to 3)	.37
	Diabetes	6 (2 to 9)	.005	5 (1 to 9)	.02	5 (1 to 9)	.02
**Variation of non–watch time^f^**							
	Referent	Reference	N/A	Reference	N/A	Reference	N/A
	Prediabetes	3 (−1 to 6)	.19	1 (−2 to 5)	.45	1 (−3 to 5)	.62
	Diabetes	13 (6 to 20)	<.001	10 (3 to 17)	.006	10 (2 to 17)	.009

^a^Model 1 covariates included sex, age, and race or ethnicity at the Framingham Heart Study health examination.

^b^Model 2 covariates included sex, age, race or ethnicity, and BMI at the Framingham Heart Study health examination.

^c^Model 3 covariates included sex, age, race or ethnicity, BMI, smoking, and alcohol drinking at the Framingham Heart Study health examination.

^d^In the analysis of daily steps as the outcome variable, we added daily smartwatch wearing time as an additional covariate in the 3 models.

^e^N/A: not applicable.

^f^The unit for variation is minute.

### Association Analyses of Variations in Watch Times With Diabetes

The participants with diabetes had larger variations in their day-to-day median values of the first watch time compared with the referents (Figure 1C and Multimedia Appendix 8). Using the 90-day window as an example, the median values of first watch times were between 6:45 AM and 8:30 AM for the participants with diabetes. In contrast, the median values of first watch times were between 7:15 AM and 7:45 AM for the referents (Figure 1C). Similar results were observed using the sampling procedures (Multimedia Appendix 8). In model 1, adjusting for age, sex, and race, on average, participants with diabetes had 12 more minutes (95% CI 6-18; *P*<.001) in the variation of the first watch time compared with the referents (Table 2). The variation in first watch time was also significantly different between the participants with prediabetes and the referents (*P*=.048; Table 2).

The participants with diabetes had a much larger variation in their median values of the last watch time compared with the referents (Figure 1D and Multimedia Appendix 9). The medians of last watch time were between 9:20 PM and 22:40 PM for the participants with diabetes; however, it was between 9:45 PM and 10 PM for the referents (Figure 1D). In model 1, on average, participants with diabetes had 6 more minutes (95% CI 2-9; *P*=.005) in the variation of last watch time compared with the referents (Table 2), adjusting for age, sex, and race. Similarly, the participants with diabetes had 13 more minutes of variation in non–watch time (95% CI 6-20; *P*<.001) compared with the referents (Table 2). The participants with prediabetes did not have significant differences in the variation of last watch time or non–watch time compared with the referents.

Adjusting for BMI in addition to age, sex, and race or ethnicity, the associations between diabetes category variables and variation in watch time variables slightly attenuated in the magnitude of associations. In model 2, participants with diabetes had 11 more minutes (95% CI 4-17; *P*=.001) in the variation of the first watch time, 5 more minutes (95% CI 1-9; *P*=.02) in the variation of the last watch time, and 10 more minutes (95% CI 3-17; *P*=.006) in the variation of the non–watch time compared with the referents (Table 2). In model 3, adjusting for alcohol consumption and smoking as additional covariates, all the estimates in both the prediabetes group and the diabetes group remained similar to model 2 (Table 2). In a sensitivity analysis, we included the number of follow-up days and daily smartwatch wearing time in addition to the covariates included in model 1. We observed a slight change in beta estimates for the first watch time, last watch time, and non–watch time (Multimedia Appendix 7).

## Discussion

### Principal Findings

To understand how daily routines function in persons with diabetes, we evaluated the associations of daily routine variables derived from a smartwatch with diabetes status in the eFHS, a community-based cohort of middle-aged to older adults. In this study, we derived several variables to reflect daily routine patterns based on smartwatch data over a long follow-up period in a home environment. We observed that the participants with diabetes, on average, walked significantly fewer steps per day compared with the referents without diabetes, adjusting for a few sets of covariates. In addition, on average, the participants with diabetes had significantly larger intraindividual variations in their daily routine patterns reflected by the first watch time, last watch time, and non–watch time compared with the referents, adjusting for the same sets of covariates.

The observation that the participants with diabetes and prediabetes walked significantly fewer daily steps compared with the referents supported the previous findings that participants with higher daily steps had a lower risk of incident diabetes [32]. In addition, the observations that the participants with diabetes had larger variations in their daily routine pattern variables than the referents are consistent with an earlier study showing that irregular daily routines are highly prevalent among adults with diabetes [11,33,34]. Previous studies have also reported that sleep abnormalities are linked to impairments in glucose homeostasis, metabolic syndrome, and diabetes [35,36]. Shift work or irregular sleep–wake cycles may increase the risk of developing diabetes [10,37,38]. Most of these previous studies collected data from laboratory tests with a small number of participants during a short study period (eg, approximately 100 participants over a few weeks) or questionnaires [10,37]. Our study used a novel approach in that we collected daily routine patterns (eg, watch times and daily steps) from smartwatches worn by 796 participants with up to 3 years of smartwatch use.

As a nested study, the eFHS was initiated at the in-person FHS health examination and completed 3 years later. To the best of our knowledge, this is the longest mHealth study with a relatively large number of middle-aged to older adult participants. However, this study was cross-sectional with respect to diabetes status as the disease status was evaluated at the time of enrollment of the eFHS. Nevertheless, the study participants were middle-aged to older adults, and their daily routine patterns were collected for >30 days and up to 3 years. Therefore, we speculated that the observations were likely to reflect their habitual daily routine patterns in adulthood. To that end, irregular daily routines (eg, daily sleep and wake-up patterns) and lower physical activity (eg, daily steps) may play important roles in the development of diabetes and are also critical in diabetes self-management.

We acknowledge that this study had several limitations. The analyses were cross-sectional with the eFHS and were ascertained after the diabetes status was evaluated at an FHS health examination; therefore, we were unable to evaluate the causal relationships between the habitual daily routine patterns and the development of diabetes. In addition, the magnitude of associations of diabetes and watch time variables was modest (<15 minutes), despite the fact that the median watch times of participants with diabetes in a 90-day follow-up had a noticeably higher day-to-day variation in the first watch time and last watch time as compared with referents. The wake-up time and bedtime were not directly measured with a standard accompanying digital survey for sleep times, although the eFHS participants were provided clear instructions that they should wear the study smartwatch daily after waking up and remove it at bedtime for charging. In addition, applying exclusions to remove low-quality data reduced the sample size from 1043 to 796. The characteristics of the excluded participants were similar to those of the included participants, indicating that the removal of participants was not likely to bias the analyses (Multimedia Appendix 4). The eFHS participants were likely to have a higher socioeconomic status, which was reflected in the lower rates of prediabetes and diabetes compared with the rest of FHS participants at the health examination; therefore, the findings in this study may not be generalizable to other populations at a higher risk for diabetes. Furthermore, the eFHS participants were middle-aged to older adults of mostly European origin from New England; thus, the generalizability of our findings to participants of other age ranges or race or ethnicities in different geographical areas remains to be studied. Moreover, the small number of participants with diabetes gave rise to wide CIs for the effect sizes. Therefore, further studies are warranted to replicate our findings.

### Conclusions

In conclusion, the eFHS is a digital cohort embedded in a traditional community-based longitudinal cohort study. Therefore, the study participants had comprehensive and accurate measures for most characteristics of their cardiovascular health. The use of the smartwatch to collect habitual physical activity and sleep behaviors in eFHS complements the traditional measurements to evaluate the role of daily routine variables in cardiovascular health. Our findings have important public health implications, as lifestyles are becoming increasingly sedentary around the globe, and the regularity of sleep behaviors is considerably disturbed by the modern environment [9,34]. On the basis of the findings of this study, health care professionals should encourage and motivate people with diabetes to increase their physical activities (eg, step counts) and maintain regular sleep behaviors, which are important for diabetes self-management. Indeed, this study has demonstrated that mHealth is a feasible and powerful approach for investigating the associations between daily routine activities and health outcomes in a traditional prospective cohort. Nevertheless, the results of this study were preliminary and hypothesis generating. Future larger mHealth studies are needed to replicate these findings. Dropout in epidemiological studies, including this study, is also an important issue in all mHealth studies. Thus, it is important to develop effective strategies to enhance adherence to improve the usefulness of mHealth in large cohort studies. In addition, advanced statistical methods are needed to account for the complex data structure and missing data of longitudinal mHealth data. With the increasing use of smartphones and the continuous improvement of mobile devices, mHealth studies will greatly enhance our understanding of the role of daily routines and lifestyle factors in the development of human diseases.

## Appendix

Multimedia Appendix 1 Flow chart of exclusion process.

Multimedia Appendix 2 Distribution of first watch time.

Multimedia Appendix 3 Distribution of last watch time.

Multimedia Appendix 4 Characteristics of 3522 participants at Framingham Heart Study health examination.

Multimedia Appendix 5 Sample sizes for 4 follow-up time windows.

Multimedia Appendix 6 The median values of daily step counts from participants in 3 diabetes categories within 90 days with sampling of the same sample size.

Multimedia Appendix 7 Association between diabetes categories and daily routine patterns measured by the smartwatch.

Multimedia Appendix 8 The median values of first watch time from participants in 3 diabetes categories within 90 days with sampling of the same sample size.

Multimedia Appendix 9 The median values of last watch time from participants in 3 diabetes categories within 90 days with sampling of the same sample size.

## Abbreviations

eFHS: electronic Framingham Heart Study
FHS: Framingham Heart Study
mHealth: mobile health
NOS: new offspring spouses
