# Supplementary material for: Comparison of Daily Routines Between Middle-aged and Older Participants With and Those Without Diabetes in the Electronic Framingham Heart Study: Cohort Study
Source: JMIR Diabetes. 2022 Jan 7;7(1):e29107. doi: 10.2196/29107 (PMC8783285; doi:10.2196/29107)
Supplement: Multimedia Appendix 1 [file diabetes_v7i1e29107_app1.docx]

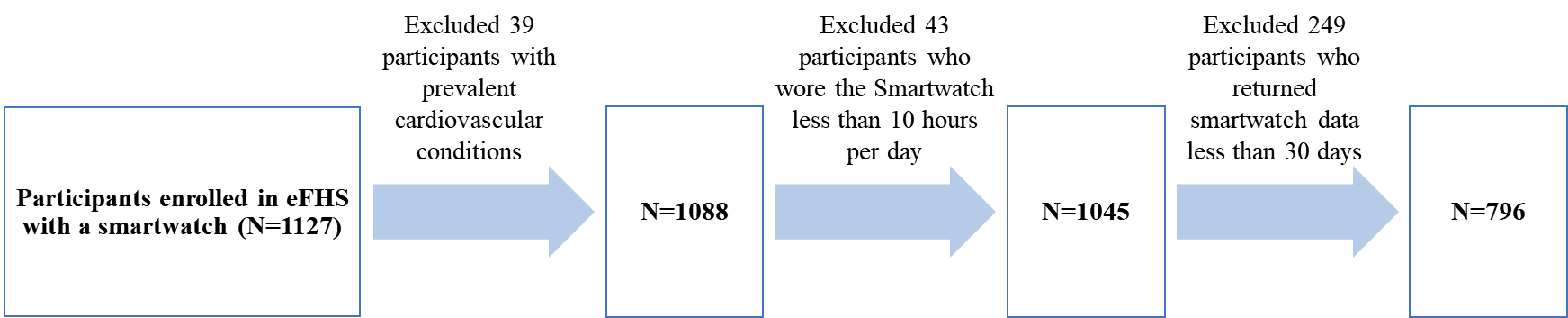


**Multimedia Appendix 1. Flow chart of exclusion process.** We excluded any watch-days with less than 10 hours because we assumed participants were more likely to follow the wearing instructions on watch-days with long wear time.
