# Supplementary material for: Comparison of Daily Routines Between Middle-aged and Older Participants With and Those Without Diabetes in the Electronic Framingham Heart Study: Cohort Study
Source: JMIR Diabetes. 2022 Jan 7;7(1):e29107. doi: 10.2196/29107 (PMC8783285; doi:10.2196/29107)
Supplement: Multimedia Appendix 2 [file diabetes_v7i1e29107_app2.docx]

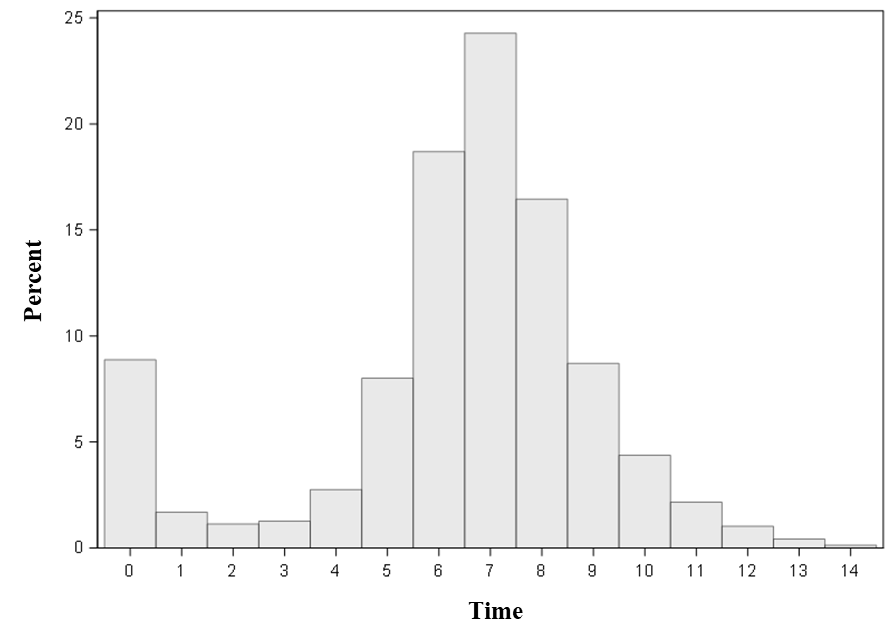


**Multimedia Appendix 2. Distribution of first watch time.** The X axis is time using 24-hours format. We defined the first watch time variable as the time when the first heart rate or step was detected by smartwatch between 4 am and noon (12 pm) on a given day. We excluded days if the first watch time was beyond this time interval.
