# Supplementary material for: Comparison of Daily Routines Between Middle-aged and Older Participants With and Those Without Diabetes in the Electronic Framingham Heart Study: Cohort Study
Source: JMIR Diabetes. 2022 Jan 7;7(1):e29107. doi: 10.2196/29107 (PMC8783285; doi:10.2196/29107)
Supplement: Multimedia Appendix 3 [file diabetes_v7i1e29107_app3.docx]

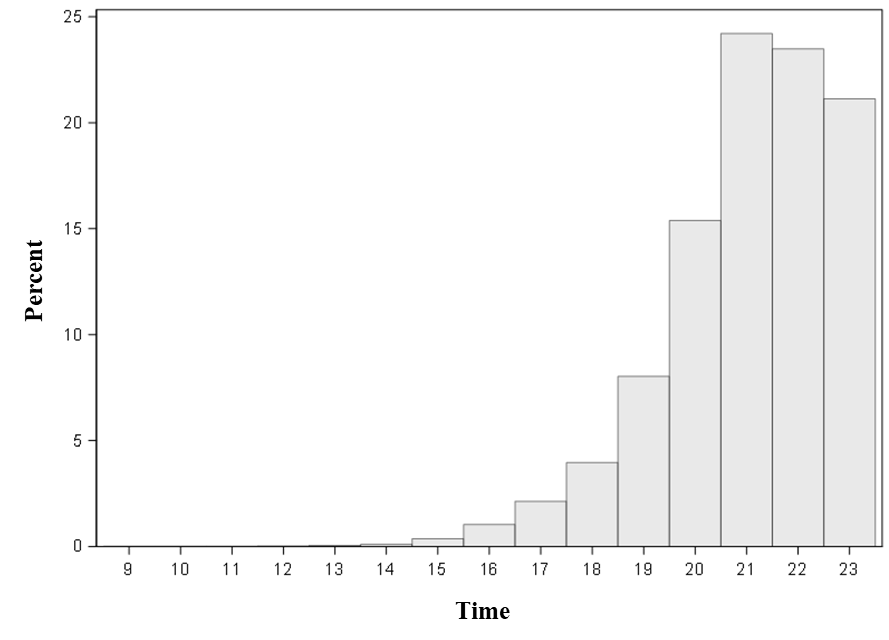
**Multimedia Appendix 3. Distribution of last watch time.** The X axis is time using 24-hours format. We defined the last-watch-time as the time when the last heart rate or step was detected by smartwatch between 7 pm and mid-night (i.e., 0:00 am), provided that the first-watch-time occurred after 4 am on the next day. We excluded days if the last watch time was beyond this time interval.
