# Supplementary material for: Comparison of Daily Routines Between Middle-aged and Older Participants With and Those Without Diabetes in the Electronic Framingham Heart Study: Cohort Study
Source: JMIR Diabetes. 2022 Jan 7;7(1):e29107. doi: 10.2196/29107 (PMC8783285; doi:10.2196/29107)
Supplement: Multimedia Appendix 4 [file diabetes_v7i1e29107_app4.docx]

**Multimedia Appendix 4. Characteristics of 3,522 participants at FHS health examination**

| **Characteristics**  **Mean ± SD or n (%)** | **FHS health examination not in eFHS**  **(n=1500)** | **eFHS not in our study**  **(n=1226)** | **Our study (n = 796)** | **Excluded participants**  **(n=331)** |
| --- | --- | --- | --- | --- |
| Age (years) | 56.8 ± 9.6 | 52.8 ± 8.9 | 52.8 ± 8.7 | 52.7 ± 8.5 |
| Women | 746 (49.7%) | 657 (53.6%) | 494 (62.1%) | 214 (64.7%) |
| Education |  |  |  |  |
| High school or less | 320 (21.3%) | 106 (8.6%) | 61 (7.6%) | 26 (7.9%) |
| Completed some college | 410 (27.3%) | 273 (22.3%) | 180 (22.6%) | 76 (23%) |
| Bachelor degree | 440 (29.3%) | 416 (33.9%) | 311 (39.1%) | 135 (40.9%) |
| Graduate or professional degree | 252 (16.8%) | 318 (25.9%) | 242 (30.4%) | 93 (28.2%) |
| BMI (kg/m^2^) | 28.9 ± 6.8 | 28.5 ± 6.0 | 28.1 ± 5.5 | 28.3 ± 5.6 |
| Diabetes status |  |  |  |  |
| Prediabetes | 435 (30.1%) | 329 (27.9%) | 209 (26.3%) | 80 (24.6%) |
| Diabetes | 186 (12.9%) | 85 (7.2%) | 41 (5.2%) | 19 (5.9%) |
