# Supplementary material for: Comparison of Daily Routines Between Middle-aged and Older Participants With and Those Without Diabetes in the Electronic Framingham Heart Study: Cohort Study
Source: JMIR Diabetes. 2022 Jan 7;7(1):e29107. doi: 10.2196/29107 (PMC8783285; doi:10.2196/29107)
Supplement: Multimedia Appendix 5 [file diabetes_v7i1e29107_app5.docx]

**Multimedia Appendix 5. Sample sizes for four follow-up time windows**

| **Diabetes categories** | **n (%)** | | | | |
| --- | --- | --- | --- | --- | --- |
|  | **Baseline**  **(n=796)** | **30 watch days**  **(n=796)** | **60 watch days**  **(n=698)** | **90 watch days**  **(n=627)** | **180 watch days**  **(n=471)** |
| Referent | 546 (100%) | 546 (100%) | 486 (89%) | 436 (80%) | 337 (62%) |
| Prediabetes | 209 (100%) | 209 (100%) | 176 (84%) | 156 (75%) | 109 (52%) |
| Diabetes | 41 (100%) | 41 (100%) | 36 (87%) | 35 (85%) | 25 (61%) |

At baseline, the study sample included 41 individuals with diabetes, 209 individuals with prediabetes and 546 individuals without either conditions.

n (%) is the number and proportion of participants at baseline, 30-, 60-, 90-, 180-day of follow up. Proportion is the ratio of the number of each follow-up and that of baseline.
