# Supplementary material for: Comparison of Daily Routines Between Middle-aged and Older Participants With and Those Without Diabetes in the Electronic Framingham Heart Study: Cohort Study
Source: JMIR Diabetes. 2022 Jan 7;7(1):e29107. doi: 10.2196/29107 (PMC8783285; doi:10.2196/29107)
Supplement: Multimedia Appendix 7 [file diabetes_v7i1e29107_app7.docx]

**Multimedia Appendix 7. Association between diabetes categories and daily routine patterns measured by the smartwatch**

|  |  | **Model 4** | |
| --- | --- | --- | --- |
| **Outcome** | **Diabetes categories** | **Mean differences**  **(95% CI)** | ***P*** |
| Daily-steps | Referent | reference |  |
|  | Prediabetes | -387 (-765, -10) | .04 |
|  | Diabetes | -1618 (-2370, -866) | <.001 |
| Absolute deviation of first watch time | Referent | reference |  |
|  | Prediabetes | 2 (-1, 6) | .20 |
|  | Diabetes | 9 (2, 16) | .01 |
| Absolute deviation of last watch time | Referent | reference |  |
|  | Prediabetes | 1 (-1, 3) | .41 |
|  | Diabetes | 4 (1, 8) | .03 |
| Absolute deviation of non-watch time | Referent | reference |  |
|  | Prediabetes | 1 (-3, 4) | .71 |
|  | Diabetes | 8 (1, 15) | .02 |

Model 4 covariates include sex, age, race/ethnicity at the FHS health examination, daily smartwatch wearing time, and follow-up days.
