# Supplementary material for: Comparison of Daily Routines Between Middle-aged and Older Participants With and Those Without Diabetes in the Electronic Framingham Heart Study: Cohort Study
Source: JMIR Diabetes. 2022 Jan 7;7(1):e29107. doi: 10.2196/29107 (PMC8783285; doi:10.2196/29107)
Supplement: Multimedia Appendix 8 [file diabetes_v7i1e29107_app8.docx]

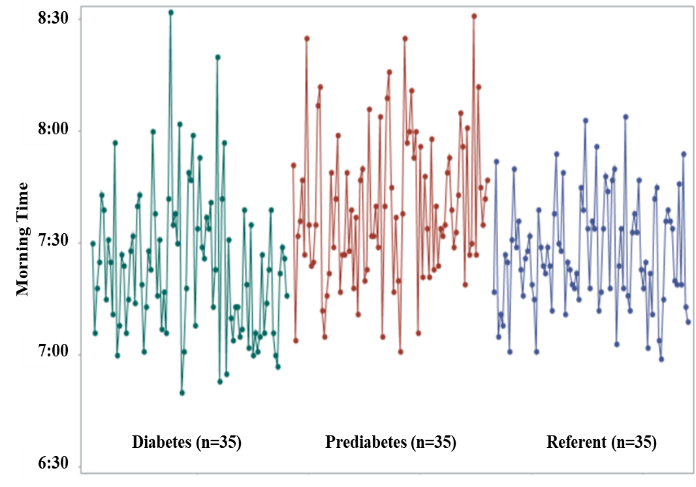

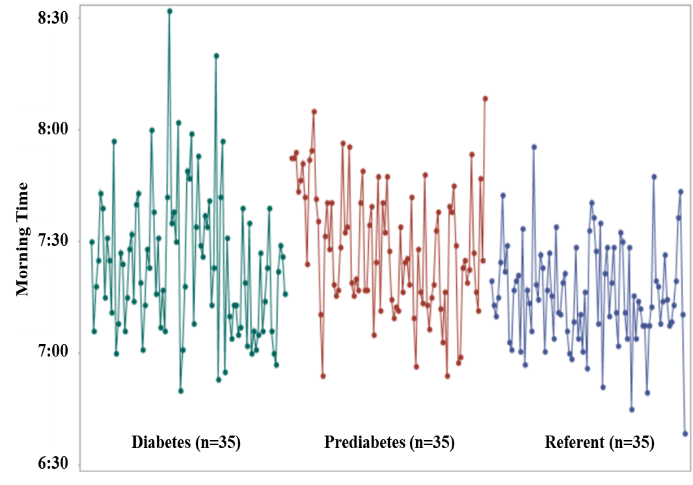

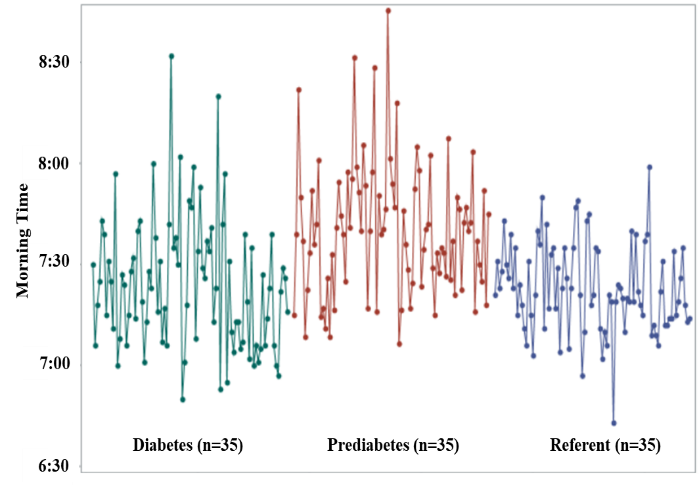

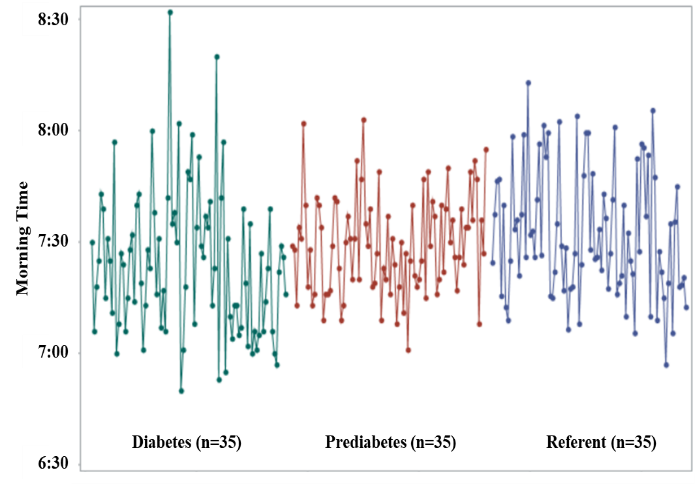

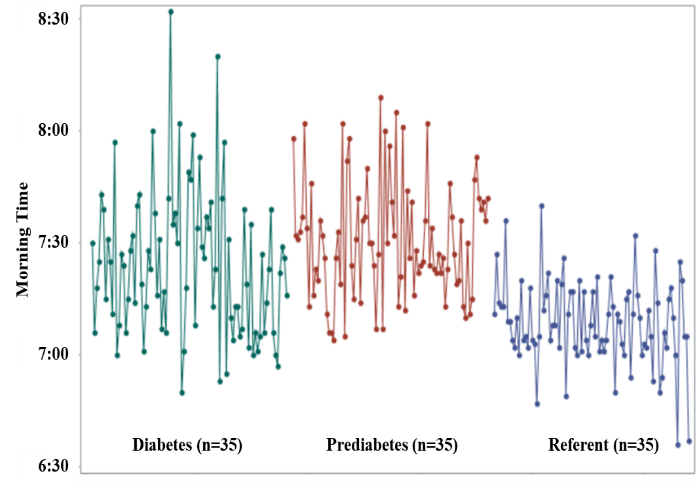


**A**

**B**

**C**

**D**

**E**

**Multimedia Appendix 8. The median values of first watch time from participants in three diabetes categories within 90 days with sampling of the same sample size.** We randomly selected 35 participants without replacement from prediabetes and referent groups. The Y axis is the median value of first-watch-time using 24-hours format.
